# Monitoring methicillin-resistant *Staphylococcus aureus* prevalence in Taiwan: a hospital-based surveillance study from 2022 to 2024

**DOI:** 10.1016/j.ijregi.2025.100702

**Published:** 2025-07-10

**Authors:** Ying-Ju Chen, Tze-Kiong Er

**Affiliations:** 1 Division of Laboratory Medicine, Asia University Hospital, Asia University, Taichung, Taiwan; 2 Department of Nursing, Asia University, Taichung, Taiwan; 3 Department of Medical Laboratory Science and Biotechnology, Asia University, Taichung, Taiwan

**Keywords:** Methicillin-resistant *Staphylococcus aureus* (MRSA), Antimicrobial resistance, Surveillance, Seasonal variation, Laboratory-based surveillance, Taiwan

## Abstract

**Objectives:**

Methicillin-resistant *Staphylococcus aureus* (MRSA) remains a significant public health concern, associated with high morbidity, increased healthcare costs, and limited treatment options due to antimicrobial resistance. Monitoring MRSA trends is essential to support effective infection prevention and control strategies in healthcare settings.

**Methods:**

This retrospective study analyzed clinical specimens tested for *Staphylococcus aureus* between January 2022 and December 2024 at a regional hospital in central Taiwan. Monthly and annual MRSA prevalence rates were calculated as the proportion of MRSA-positive isolates. A chi-square test of independence was used to assess the statistical significance of prevalence differences across years.

**Results:**

A total of 1,824 *Staphylococcus aureus* isolates were identified during the study period, including 287 MRSA cases in 2022 (48.9%), 281 in 2023 (42.3%), and 278 in 2024 (48.5%). The chi-square test revealed a statistically significant difference in annual MRSA prevalence (χ² = 6.94, *P* = 0.031), indicating a meaningful variation in resistance patterns across years.

**Conclusion:**

MRSA prevalence fluctuated over the 3-year period, with a temporary decline in 2023 followed by a rebound in 2024. These findings underscore the importance of continuous surveillance and adaptable infection control strategies to address evolving antimicrobial resistance in hospital environments.

## Highlights

• Methicillin-resistant *Staphylococcus aureus* (MRSA) surveillance conducted in a Taiwanese hospital from 2022 to 2024.
• Annual MRSA rates fluctuated: 48.9% (2022), 42.3% (2023), 48.5% (2024).
• MRSA prevalence peaked during summer in 2022 and 2023.
• No consistent decline in MRSA burden was observed over 3 years.
• Data support ongoing MRSA monitoring and adaptive infection control.

## Introduction

Methicillin-resistant *Staphylococcus aureus* (MRSA), first identified in the 1960s, has become a major cause of both healthcare- and community-associated infections globally [1]. It can lead to a wide range of clinical conditions, from mild skin and soft tissue infections to severe illnesses such as pneumonia and bloodstream infections, with outcomes influenced by patient comorbidities and infection sites [1]. MRSA poses significant clinical and economic challenges due to increased morbidity, prolonged hospital stays, and elevated healthcare costs [2,3]. For example, a nationwide study in Japan estimated that MRSA infections contributed to over US$2 billion in excess healthcare expenditures and more than 14,000 deaths annually [4]. MRSA resistance to β-lactam antibiotics is primarily mediated by the acquisition of the mecA gene, which encodes penicillin-binding protein 2a, reducing the efficacy of methicillin and related agents. Additional resistance may arise through β-lactamase production [5].

In Taiwan, the prevalence of methicillin-resistant *Staphylococcus aureus* (MRSA) among *Staphylococcus aureus* isolates has remained high since the 1990s, with several studies reporting sustained levels exceeding 50% [6,7]. Infection prevention efforts typically include patient isolation, enhanced environmental cleaning, and decolonization with intranasal mupirocin. Early identification and timely implementation of infection control measures are essential to prevent transmission [2]. One study showed genetic similarity between MRSA strains found in hospital environments and those from clinical infections, highlighting the potential role of environmental reservoirs in nosocomial spread [8].

Given the global and local impact of MRSA, regular institutional surveillance is essential for tracking trends and guiding targeted infection control strategies. This retrospective study analyzed MRSA prevalence among clinical *Staphylococcus aureus* isolates at a regional hospital over a 3-year period (2022–2024). By examining annual and monthly prevalence patterns, the study aims to provide insight into local MRSA dynamics and support ongoing antimicrobial resistance (AMR) management efforts.

## Materials and methods

### Study design and setting

This retrospective observational study was conducted at a regional hospital in central Taiwan with over 450 beds, offering comprehensive acute and specialized medical care. Laboratory-based surveillance data were collected from January 1, 2022, to December 31, 2024, focusing on clinical specimens yielding *Staphylococcus aureus*. Isolates were recovered from various sources, including blood, urine, respiratory secretions, wound swabs, and other routine clinical samples. The primary objective was to evaluate the prevalence of MRSA among all *Staphylococcus aureus* isolates across the 3-year period. This study was approved by the Institutional Review Board of China Medical University Hospital (IRB No. CMUH114-REC3-110). As only anonymized laboratory data were used, the requirement for informed consent was waived.

### Data collection

Data were extracted from the hospital’s laboratory information system. For each month, the total number of *Staphylococcus aureus* isolates and the number identified as MRSA were recorded. To minimize confounding effects, duplicate isolates from the same patient were excluded. According to institutional records, there were no significant changes in infection prevention and control policies, sampling protocols, or critical care capacity during the study period. This procedural consistency enhances the comparability of MRSA prevalence across years and reduces the risk of major confounding factors.

### Bacterial identification and antimicrobial susceptibility profiling using the phoenix 100 automated system

Clinical specimens were initially inoculated onto eosin methylene blue (EMB) agar and blood agar (BAP) and incubated at 35°C for 18–24 hours. Bacterial identification and susceptibility testing were performed using the Phoenix 100® automated system (BD Diagnostics, Sparks, MD, USA), which integrates modified conventional, fluorogenic, and chromogenic substrates. Testing utilized the BD NMIC/ID-411, PMIC/ID-95, and SMIC/ID-2 panels, and software versions V6.21A and V6.35A were employed during the study period. Pure colonies were suspended in ID broth, adjusted to a 0.5 McFarland turbidity standard using a nephelometer (BD PhoenixSpec™ nephelometer), and tested per the manufacturer’s instructions [9]. Antimicrobial susceptibility was interpreted as susceptible (S), intermediate (I), or resistant (R) based on Clinical and Laboratory Standards Institute guidelines.

### Data analysis

Monthly and annual MRSA prevalence rates were calculated as the proportion of MRSA-positive isolates relative to the total number of *Staphylococcus aureus* isolates, expressed as percentages. A chi-square test of independence was performed using IBM SPSS Statistics for Windows, Version 31.0 (IBM Corp., Armonk, NY, USA) to assess whether the differences in MRSA prevalence across the 3 years were statistically significant. A *P*-value of <0.05 was considered statistically significant.

## Results

Between January 1, 2022, and December 31, 2024, a total of 1,824 *Staphylococcus aureus* isolates were identified from clinical specimens. Of these, 587 were collected in 2022, 664 in 2023, and 573 in 2024. The number of methicillin-resistant isolates was 287 (48.9%) in 2022, 281 (42.3%) in 2023, and 278 (48.5%) in 2024, indicating year-to-year variation in MRSA prevalence.

**Figure 1:**
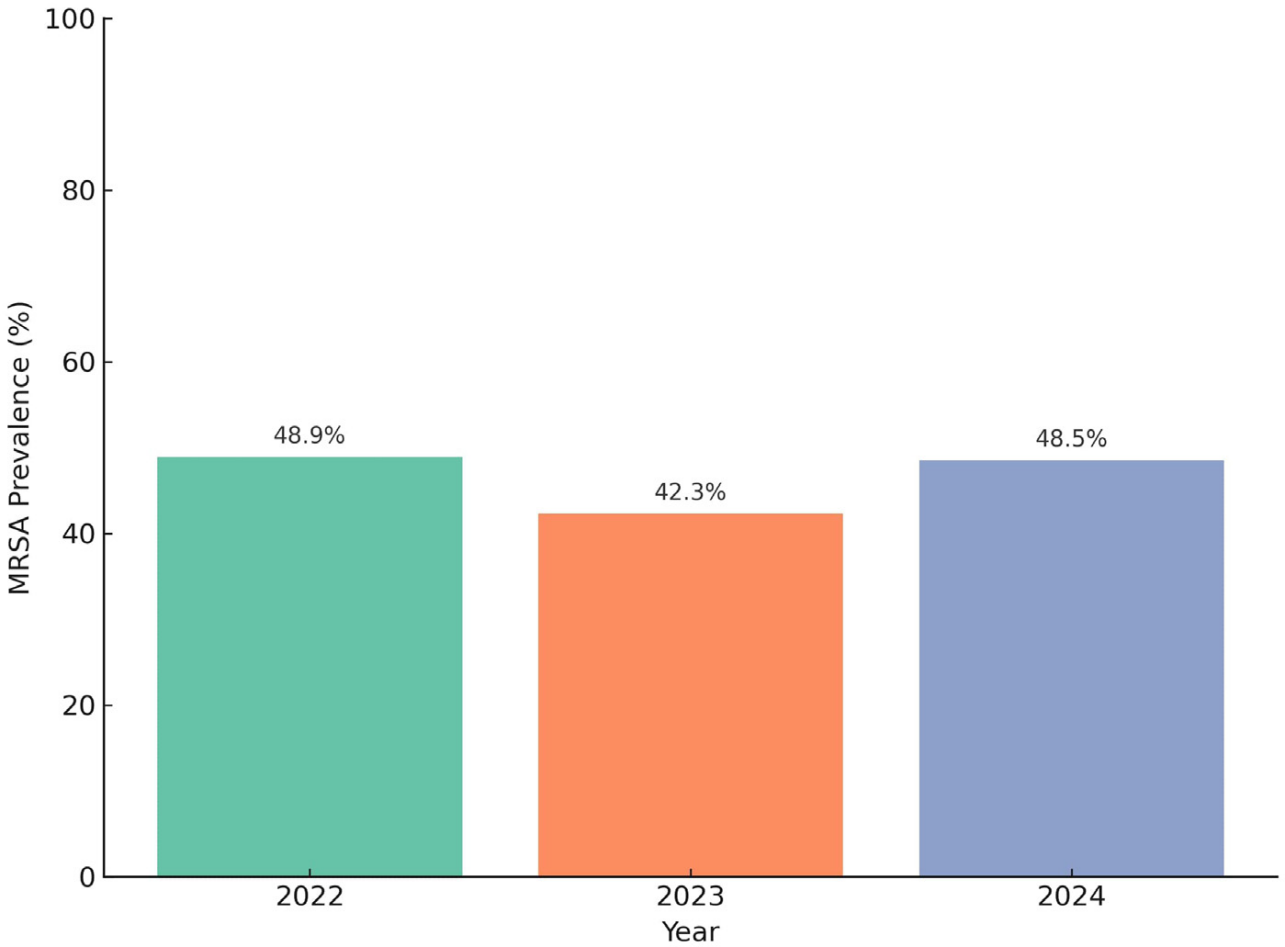
Annual MRSA prevalence among *Staphylococcus aureus* isolates from 2022 to 2024. This bar chart presents the percentage of *Staphylococcus aureus* isolates that were MRSA each year. The prevalence was 48.9% in 2022, declined to 42.3% in 2023, and increased again to 48.5% in 2024. A chi-square test of independence showed statistically significant variation across the 3 years (χ² = 6.94, *P* = 0.031). MRSA, methicillin-resistant *Staphylococcus aureus*.

A Chi-square test of independence showed that the annual differences in MRSA prevalence were statistically significant (χ² = 6.94, *P* = 0.031), confirming that the observed shifts are unlikely to be due to random variation. While a decline occurred in 2023, the rate rebounded in 2024, suggesting a fluctuating rather than linear trend. These findings underscore the importance of continuous local surveillance to track antimicrobial resistance dynamics (Figure 1).

**Figure 2:**
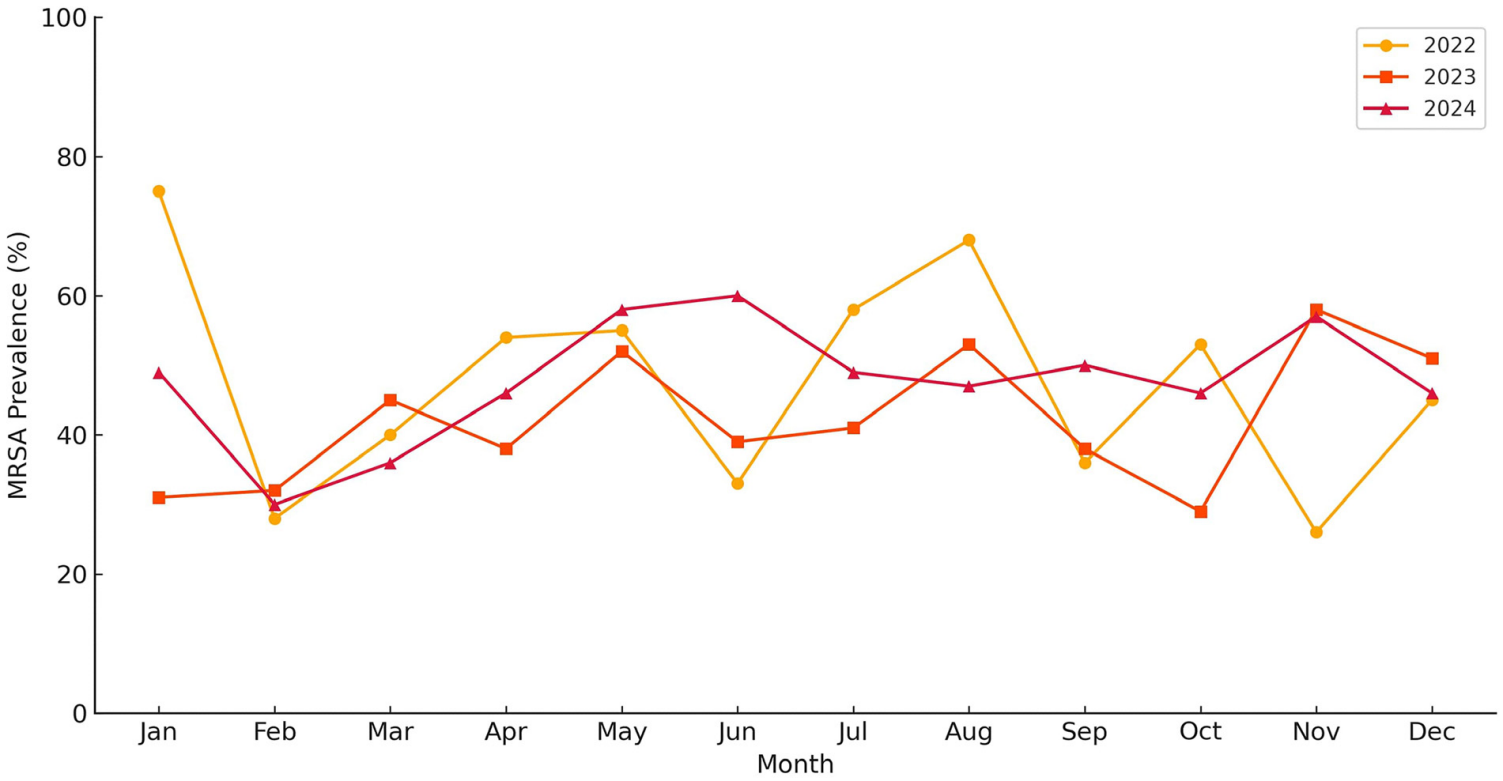
Monthly MRSA prevalence among *Staphylococcus aureus* isolates from 2022 to 2024. This line chart illustrates the monthly variation in MRSA prevalence over a 3-year period. Data represent the proportion of MRSA-positive isolates among total *Staphylococcus aureus* isolates, as recorded in the hospital’s laboratory surveillance system. Elevated MRSA prevalence was noted during summer months in 2022 and 2023, while a different distribution pattern was observed in 2024. MRSA, methicillin-resistant *Staphylococcus aureus*.

Monthly trends demonstrated notable variability (Figure 2). In 2022 and 2023, MRSA prevalence peaked during the summer months, particularly in July and August, with values exceeding 50%. The highest single-month prevalence was recorded in January 2022 (75.0%). In contrast, the peak months in 2024 occurred in May and November, indicating a shift from the earlier seasonal pattern. While these fluctuations may be influenced by environmental conditions or operational dynamics, no consistent seasonal trend was observed across all 3 years. These findings underscore the importance of high-resolution, time-based surveillance to inform timely infection control interventions.

## Discussion

This study presents an updated analysis of MRSA prevalence trends over a 3-year period at a regional hospital in central Taiwan. While a temporary decrease was observed in 2023, the overall trend was fluctuating, with prevalence rebounding in 2024. These variations may reflect a multifactorial interplay of infection control practices, antimicrobial stewardship initiatives, seasonal effects, and institutional factors. The findings underscore the importance of continuous surveillance and flexible prevention strategies tailored to local epidemiology.

MRSA remains a globally significant healthcare-associated pathogen due to its resistance to multiple antibiotic classes, particularly β-lactams, which complicates treatment and infection management [10]. It is associated with a broad spectrum of clinical manifestations, ranging from skin and soft tissue infections to severe systemic conditions such as pneumonia and bacteremia. Infections can result in serious complications, including sepsis, myocardial infarction, and heart failure, especially in vulnerable patients [[11], [12], [13]].

Although MRSA prevalence in our hospital showed fluctuations—declining from 48.9% in 2022 to 42.3% in 2023 before rising again to 48.5% in 2024—these changes may still hold clinical relevance. In contrast, a review from Nigeria reported a steady rise from 18.3% in 2009 to 42.3% in 2013, emphasizing resistance to commonly used, low-cost oral antibiotics and signaling an expanding threat across regions [14]. International comparisons further illustrate MRSA’s variability. A 2017 study in Thailand found a prevalence of 17%, with higher rates in hospital-acquired infections and among patients with chronic conditions [15]. Conversely, research from Eritrea revealed a high MRSA rate of 72%, alongside resistance to vancomycin, erythromycin, and gentamicin, highlighting major treatment challenges [16]. Our 2022 prevalence closely aligns with a 5-year study in Macau, which reported 45.7% for the same year. However, while Macau showed a continuous rise from 2017 to 2022, our data displayed a fluctuating pattern, reflecting differences in healthcare practices and surveillance systems [17]. More recently, a study from a Saudi maternity and children’s hospital reported a 45.4% MRSA prevalence, primarily in skin and wound infections, with all isolates remaining sensitive to vancomycin and linezolid [18]. In the U.S., MRSA accounted for 44% of antimicrobial-resistant infections in 2022, with a concerning resurgence in hospital-onset cases during the COVID-19 pandemic years (2020–2021), despite prior declines [19]. A meta-analysis from Qatar showed a pooled MRSA prevalence of 52.4% among resistant isolates—substantially higher than global averages for high-income countries—further underscoring the importance of tailored infection control policies in Gulf nations [20]. Together, these studies highlight the global burden of MRSA and the urgent need for sustained surveillance, rigorous infection control measures, and effective antimicrobial stewardship.

The seasonal variation observed in our study—marked by higher MRSA prevalence during the summer months of 2022 and 2023, followed by a temporal shift in 2024—suggests that environmental or operational factors may influence MRSA transmission dynamics. This pattern aligns with findings from Rhode Island Hospital, which reported increased MRSA infection rates during summer and autumn, particularly among pediatric patients [21]. Similarly, studies in U.S. nursing homes documented seasonal peaks in MRSA colonization during the spring, affecting both residents and environmental surfaces [22]. Additionally, prior research has linked cooler monthly average temperatures to increased MRSA prevalence at both individual and household levels [23]. These observations collectively support the hypothesis that environmental conditions—such as temperature and humidity—play a role in MRSA transmission. Consequently, they highlight the importance of seasonally responsive infection surveillance and prevention strategies in both acute and long-term care settings.

The temporary decline in MRSA prevalence observed in 2023 may be associated with short-term improvements in infection control practices, such as increased hand hygiene compliance, the implementation of targeted decolonization protocols, or enhanced environmental cleaning. The recurring mid-year increases in 2022 and 2023 could potentially reflect seasonal factors, including warmer temperatures that may promote higher skin colonization rates or fluctuations in hospital activity, such as elective admissions. These observations highlight the multifactorial nature of MRSA transmission dynamics and support the need for ongoing, seasonally responsive infection control strategies.

This study has several limitations. First, as a retrospective, single-center analysis, the findings may not be fully generalizable to other healthcare settings with differing patient populations, infection control policies, or resource availability. Second, the lack of detailed patient-level clinical data precluded the evaluation of individual risk factors such as underlying comorbidities, prior antibiotic exposure, or length of hospitalization. Third, molecular typing of MRSA isolates was not conducted, which limited the ability to assess potential clonal relationships or transmission dynamics within the hospital. Although granular infection prevention and control policy data were unavailable, institutional records confirmed that infection control practices, sampling protocols, and critical care capacity remained stable throughout the study period, which may mitigate—but does not eliminate—the risk of confounding. Despite these limitations, the study is strengthened by its robust sample size, use of standardized microbiological methods, and consistent data collection over a 3-year period.

In summary, this 3-year surveillance study revealed statistically significant year-to-year and seasonal variations in MRSA prevalence, although no sustained downward trend was observed. These fluctuations may reflect complex interactions between environmental factors, patient demographics, and institutional practices. The findings underscore the critical need for continuous antimicrobial resistance surveillance, real-time data reporting, and rapid implementation of infection control interventions. Monitoring resistance trends on both annual and monthly scales allows for the early detection of outbreaks, the identification of lapses in infection prevention, and the refinement of antimicrobial stewardship programs.

## Conclusion

Over the 3-year period from 2022 to 2024, MRSA prevalence at our institution exhibited marked temporal fluctuations without a consistent downward trend. These findings underscore the persistent burden of MRSA and highlight the ongoing need for comprehensive infection prevention strategies in hospitals. Key measures should include sustained microbiological surveillance, timely implementation of decolonization protocols, and robust antimicrobial stewardship to reduce MRSA-related morbidity, mortality, and healthcare resource utilization.

## Declarations of competing interest

The authors have no competing interests to declare.

## References

[1] Lee A.S., de Lencastre H., Garau J., Kluytmans J., Malhotra-Kumar S., Peschel A. (2018). Methicillin-resistant Staphylococcus aureus. Nat Rev Dis Primers.

[2] Samuel P., Kumar Y.S., Suthakar B.J., Karawita J., Sunil Kumar D.S., Vedha V. (2023). Methicillin-resistant Staphylococcus aureus colonization in intensive care and burn units: a narrative review. Cureus.

[3] Tong S.Y.C., Davis J.S., Eichenberger E., Holland T.L., Fowler V.G. (2015). Staphylococcus aureus infections: epidemiology, pathophysiology, clinical manifestations, and management. Clin Microbiol Rev.

[4] Uematsu H., Yamashita K., Kunisawa S., Fushimi K., Imanaka Y. (2017). Estimating the disease burden of methicillin-resistant Staphylococcus aureus in Japan: retrospective database study of Japanese hospitals. PLoS One.

[5] Abebe A.A., Birhanu AG. (2023). Methicillin resistant Staphylococcus aureus: molecular mechanisms underlying drug resistance development and novel strategies to combat. Infect Drug Resist.

[6] Ho M., McDonald L.C., Lauderdale T.L., Yeh L.L., Chen P.C., Shiau YR. (1999). Surveillance of antibiotic resistance in Taiwan, 1998. J Microbiol Immunol Infect.

[7] Chen C.J., Huang YC. (2014). New epidemiology of Staphylococcus aureus infection in Asia. Clin Microbiol Infect.

[8] Yu C.H., Shen S., Huang K.A., Huang YC. (2022). The trend of environmental and clinical methicillin-resistant Staphylococcus aureus in a hospital in Taiwan: impact of USA300. J Microbiol Immunol Infect.

[9] O'Hara CM. (2006). Evaluation of the Phoenix 100 ID/AST system and NID panel for identification of Enterobacteriaceae, Vibrionaceae, and commonly isolated nonenteric gram-negative bacilli. J Clin Microbiol.

[10] Tacconelli E., Carrara E., Savoldi A., Harbarth S., Mendelson M., Monnet D.L. (2018). Discovery, research, and development of new antibiotics: the WHO priority list of antibiotic-resistant bacteria and tuberculosis. Lancet Infect Dis.

[11] Otarigho B., MO Falade (2023). Computational screening of approved drugs for inhibition of the antibiotic resistance gene mecA in methicillin-Resistant Staphylococcus aureus (MRSA) strains. BioTech (Basel).

[12] Khairullah A.R., Rehman S., Agus Sudjarwo S., Effendi M.H., Chasyer Ramandinianto S., Aega Gololodo M. (2022). Detection of mecA gene and methicillin-resistant Staphylococcus aureus (MRSA) isolated from milk and risk factors from farms in Probolinggo. Indonesia. F1000Res.

[13] Inagaki K., Lucar J., Blackshear C., Hobbs CV. (2019). Methicillin-susceptible and methicillin-resistant Staphylococcus aureus bacteremia: nationwide estimates of 30-day readmission, in-hospital mortality, length of stay, and cost in the United States. Clin Infect Dis.

[14] Abubakar U., Sulaiman SAS. (2018). Prevalence, trend and antimicrobial susceptibility of methicillin resistant Staphylococcus aureus in Nigeria: a systematic review. J Infect Public Health.

[15] Waitayangkoon P., Thongkam A., Benjamungkalarak T., Rachayon M., Thongthaisin A., Chatsuwan T. (2020). Hospital epidemiology and antimicrobial susceptibility of isolated methicillin-resistant Staphylococcus aureus: a one-year retrospective study at a tertiary care center in Thailand. Pathog Glob Health.

[16] Garoy E.Y., Gebreab Y.B., Achila O.O., Tekeste D.G., Kesete R., Ghirmay R. (2019). Methicillin-resistant Staphylococcus aureus (MRSA): prevalence and antimicrobial sensitivity pattern among patients-A multicenter study in Asmara, Eritrea. Can J Infect Dis Med Microbiol.

[17] Xing A., Ng H.M., Jiao H., Li K., Ye Q. (2024). The prevalence, epidemiological, and molecular characterization of methicillin-Resistant Staphylococcus aureus (MRSA) in Macau (2017–2022). Microorganisms.

[18] Almutairi H., Albahadel H., Alhifany A.A., Aldalbahi H., Alnezary F.S., Alqusi I. (2024). Prevalence and antimicrobial susceptibility pattern of methicillin-resistant Staphylococcus aureus (MRSA) at a maternity and children hospital in Saudi Arabia: a cross-sectional study. Saudi Pharm J.

[19] Wolford H., McCarthy N.L., Baggs J., Hatfield K.M., Maillis A., Olubajo B. (2025). Antimicrobial-resistant infections in hospitalized patients. JAMA Netw Open.

[20] Abu El-Ruz R.A., Masoud O.A., Ibrahim A.A., Chivese T., Zughaier S.M. (2025). The epidemiology of antimicrobial resistant bacterial infection in Qatar: a systematic review and meta-analysis. J Infect Public Health.

[21] Mermel L.A., Machan J.T., Parenteau S. (2011). Seasonality of MRSA infections. PLoS One.

[22] Cassone M., Mantey J., Gontjes K.J., Lansing B.J., Gibson K.E., Wang J. (2021). Seasonal patterns in incidence and antimicrobial resistance of common bacterial pathogens in nursing home patients and their rooms. Front Public Health.

[23] Mork R.L., Hogan P.G., Muenks C.E., Boyle M.G., Thompson R.M., Morelli J.J. (2018). Comprehensive modeling reveals proximity, seasonality, and hygiene practices as key determinants of MRSA colonization in exposed households. Pediatr Res.

